# An evaluation of the use of caries risk/susceptibility assessment in an undergraduate dental curriculum

**DOI:** 10.3389/froh.2023.1290713

**Published:** 2024-01-29

**Authors:** Melanie Nasseripour, Adam Hasan, Liz Chapple, Anusha Chopra, Lucy Cracknell, Zahraa Maiter, Aviijit Banerjee

**Affiliations:** ^1^ Centre for Dental Education, Faculty of Dentistry, Oral and Cranio-Facial Sciences, King’s College London, London, United Kingdom; ^2^ OHI ltd., Birmingham, United Kingdom; ^3^ Centre for Oral, Clinical & Translational Sciences, Faculty of Dentistry, Oral and Cranio-Facial Sciences, King’s College London, London, United Kingdom

**Keywords:** dental caries susceptibility, risk assessment, oral health, undergraduate, curriculum

## Abstract

There has been a paradigm shift in patient care with regards to delivering better oral health, towards a team-delivered, person-focused, risk-related model that is known as minimum intervention oral care (MIOC). Student skills should be developed within the undergraduate dental curricula to ensure that graduating dentists and other oral healthcare team members are able to provide phased personalised care plans alongside behavioural management support to patients/caregivers to prevent and manage oral disease in the long term. The purpose of this study is to establish that using an adjunctive caries risk/susceptibility assessment (CRSA) technology (PreViser) had an impact on the behaviour, perception, and knowledge of dental undergraduate students and their clinical teachers, regarding the benefits of such an oral health assessment in the management of patients. Four cohorts of students who did not have exposure to the caries risk susceptibility software were compared with those who did. This study was conducted using a mixed methods approach using a convergent parallel design consisting of collecting quantitative data through questionnaires presented to four cohorts of Year 4 dental students (*n* = 150 per cohort) and their clinical teachers (*n* = 10) and qualitative data from structured interviews with students (*n* = 5) and teachers (*n* = 7) with suitable statistical analysis and interpretation.

**Results:**

Generally, the items that exhibited statistical significance, when reviewed, showed better behaviour, perception, and knowledge towards CRSA in the Group C (BDS4-22T1) cohort in comparison with the Group A (BDS3-20T2) cohort. The Group D (BDS4-22T2) students felt more confident using the PreViser as a CRSA tool. When comparing the Group C and Group D data, we note that the students from the Group C cohort were more likely to carry out a diet analysis for their patients and were less likely to be negatively impacted by time constraints compared with the Group D students. Both cohorts were equally confident in using the PreViser for CRSA. From a qualitative perspective, although competence and confidence appeared high, the students and teachers acknowledged that they would need more support to use it chairside. The main barrier listed to using PreViser rested in the fact that clinical teachers either preferred their own ways of assessing or did not know how to use the tool and therefore did not encourage using it. Those who did use PreViser highlighted that it was straightforward to use and was a systematic approach, enabling communication with the patients as there is ‘evidence’ to back up the clinical recommendations.

**Conclusion:**

The cumulative benefit of training and use (even limited) had an impact on the students' knowledge, competence, and confidence regarding CRSA, ultimately facilitating the process of teaching and assisting them in effectively implementing CRSA. The importance of CRSA became more evident immediately following the training. Further research is suggested to understand the factors influencing student behaviour, perception, and knowledge regarding CRSA with the aim to make recommendations on a preferable approach and tool to help streamline CRSA education.

## 1 Introduction

Considering the preventable nature of behaviour-related oral disease (e.g., dental caries), the provision of clinical treatment as a sole measure of outcome success is dated and inappropriate, with a preventive, long-term approach to maintaining oral health now being recommended (1). Risk/susceptibility assessment facilitates targeted prevention by enabling and supporting conversations with patients or caregivers regarding their patient's combination of risk factors impacting their future oral health outcomes. Furthermore, identifying relevant changes and implementing suitable preventive measures, both within the dental surgery and at home (self-care), to address/minimise these risk factors, can contribute to achieving optimal long-term oral health outcomes.

There has been a paradigm shift in patient care within delivering better oral health, towards a team-delivered, person-focused, risk-related model, that is minimum intervention oral care (MIOC) (2–5). Person-focused care requires educating dental professionals on oral health risk/susceptibility factor assessment, that is, the risk/susceptibility factors for caries, periodontal disease, tooth surface loss, and oral cancer (6). By applying this assessment, a structured, phased, personalised care plan can be developed with an engaged, motivated patient/caregiver, to change behaviours and achieve successful long-term oral healthcare maintenance.

The undergraduate dental curricula should prioritise the development of student skills to ensure that graduating dentists and other members of the oral healthcare team are capable of providing personalised care plans based on person-focused oral health risk/susceptibility assessments, as well as offering behavioural management support to patients.

The Faculty of Dental, Oral and Cranio-facial Sciences, King's College London (FoDOCS), a UK teaching institution, has a long history of embedding Minimum Intervention Oral Health Care including Caries Risk/Susceptibility Assessment (CRSA) throughout its 5-year undergraduate curriculum of the Bachelor of Dental Sciences (BDS) programme. From an educational perspective, the learning outcomes are mapped to the registration outcomes set by the regulatory body for the UK dental profession (7), requiring dentists to ‘evaluate the health risks of diet, drugs and substance misuse, and substances such as tobacco and alcohol on oral and general health and provide appropriate advice and support’.

Teaching/education must include evaluating the learning process and its effects on both student clinical practice and patient health. Continuous assessment of student behaviour, perception, and knowledge of CRSA from 2017 onwards informed the changes which were implemented within the current dental curriculum. The outcome of these assessments, in particular, highlighted the usefulness of a systematic approach for evaluating the risk/susceptibility to oral health at the chairside in clinics to support students in improving clinical outcomes for their patients. The choice of the online PreViser technology was informed by the need to have a comprehensive oral disease risk/susceptibility assessment tool for caries, periodontology, oral cancer, and tooth surface loss, which was applicable in an undergraduate academic environment.

PreViser is an online tool used to evaluate the risk/susceptibility to oral diseases, as well as assess oral health which, up to 2023, was supplied in the United Kingdom by OHI Ltd, a joint venture with the University of Birmingham. Currently, PreViser is available worldwide through PreViser Corporation. Since 2017, oral disease risk assessment has been embedded in undergraduate training at the University of Birmingham Dental School, and the use of PreViser formed a required competency. In the United Kingdom, 845 dentists performed 160,000 assessments using the Denplan PreViser Patient Assessment (DEPPA) version of the software. In the United States, PreViser is owned by an insurance company, NE Delta Dental, that promotes an (8) approach to patient care and primarily uses PreViser as the entry into enhanced benefits for specific conditions (https://www/healththroughoralwellness.com). Over 1 million PreViser risk assessments have been completed across the United States, and more than 150 schools/universities/colleges in 43 states are registered users of PreViser Clinical Suite (source: PreViser Corporation).

### 1.1 Aims and objectives

The purpose of this study is to identify whether using adjunctive CRSA technology (PreViser) had an impact on the behaviour, perception, and knowledge of dental undergraduate students and their clinical teachers regarding the benefits of oral health assessment in the management of patients.

The working hypothesis was that the impact of using PreViser would enhance student behaviour, perception, and knowledge of oral health risk assessment in the management of patients.

Having assessed the feasibility of implementing CRSA technology at FoDOCS clinical facility in Guy's and St Thomas’ Hospital Trust (GSTT), these results would help inform future changes in the broader curriculum reviews regarding the advancement of Oral Health Risk/Susceptibility Assessment using such adjunctive technology.

## 2 Materials and methods

This study was conducted using a mixed methods approach with a convergent parallel design; quantitative data (from questionnaires) and qualitative data (from interviews and focus groups) were collected and analysed to determine whether student behaviour, perception, and/or knowledge had changed. The areas of convergence or divergence between the qualitative and quantitative results should be discussed. The quantitative and qualitative data were collected via questionnaires and interviews, enabling us to establish a detailed and accurate picture of the characteristics and behaviours of a particular population (here, students) towards a specific topic (here, CRSA). The ethical approval was obtained from King's College London Research Ethics Committee (ref: LRS-20/21-20542).

### 2.1 Description of participants

The data collected from the research project consisted of two groups of participants: the student group and the clinical teacher group.

#### 2.1.1 Student group

The student cohort using PreViser was the BDS4 academic year 2021–2022 cohort. We looked at their responses to the student questionnaire, before (BDS4-22T1) and after (BDS4-22T2) the PreViser training and use. We also compared their questionnaire responses to an equivalent cohort in academic year 2019–2020 as explained in Table 1:

• BDS4-22T1/Group C with BDS3-20T2/Group A.
• BDS4-22T2/Group D with BDS4-20T2/Group B.

**Table 1:** Abbreviations and participant denominations. T1 refers to the start of the academic year, and T2 refers to the end.

Abbreviation	Student cohort description	Equivalence	CRSA education and training overview		
			Similarities	Differences	
BDS3-20T2/Group A	BDS3 cohort at the end of the academic year 2019-2020	As there is no teaching or clinics over the summer we can consider that BDS3-20T2 are equivalent to BDS4 at start of the year in T1	• same profile (age/gender/clinics) • same numbers in the cohort and participating in the study • same curriculum in Cons/Mi	No PreViser-specific training or use	
BDS4-20T2/Group B	BDS4 cohort at the end of the academic year 2019–2020			No PreViser-specific training or use	
BDS4-22T1/Group C	BDS4 cohort at the start of the academic year 2021–2022, and therefore before the start of the study.			PreViser-specific training as outlined	entire BDS3 clinical experience impacted by COVID-19 although the rest of the curriculum was delivered
BDS4-22T2/Group D	BDS4 cohort at the end of the academic year 2021–2022, and therefore after the end of the study.			PreViser-specific training as outlined and used PreViser in CPC clinics	

In addition, the BDS4-22T2 students were invited to attend online interviews conducted through Microsoft Teams (MS Teams) to discuss their behaviour, perception, and knowledge of CRSA, with a specific focus on PreViser. Due to practical logistical challenges, we had to interview each member of the student focus group separately (clinic timetabling clashing with the ability for all to attend the same session).

#### 2.1.2 Clinical teacher group

The group of clinical teachers questioned on PreViser was the Undergraduate Clinical teachers who would have had direct clinical teaching of the BDS4-22 cohort throughout academic year 2021–2022.

We looked at their responses to the clinical teacher survey before and after the PreViser training and use.

In addition, the clinical teachers were invited to attend online focus groups using MS Teams to discuss their behaviour, perception, and knowledge of CRSA, with a particular focus on PreViser. Due to practical logistical challenges (teaching timetabling clashing with the ability for all to attend the same session), we had to break the group into three separate focus group discussions.

#### 2.1.3 Intervention

We introduced PreViser to students in the 4th year of the programme (BDS4-22 students, *n* = 150) and their Care Planning Clinics (CPC) teachers (*n* = 10). All participants were calibrated to use PreViser as an Oral Health Risk Assessment tool (see below section on the training of teachers and students) to support care planning for a duration of 5 months starting 1 November 2021 to 30 April 2022. There were a total of 102 PreViser assessments by students during this period of time.

The details of the teacher and student training to calibrate their proficiency in using PreViser:

The students in BDS3 and BDS4 get two formal lectures and seminars each year on Oral Health Risk/Susceptivity Assessment including specifically CRSA in Years 3, 4, and 5 of their undergraduate curriculum with PreViser reviewed among other tools. In the summer of 2018, the clinical staff were made aware of PreViser as part of their training to become King's College London Behaviour Change champions, and all clinical teachers are involved in delivering the Conservative and Minimal Invasive dentistry (Cons/MI) seminars which also cover CRSA tools including PreViser. In preparation for the start of the PreViser pilot study, we implemented the following training for the students and teachers:

August–September: Prior to the start of the PreViser pilot study at care planning clinics in October:

• Materials posted on the Keats BDS4 year group page (virtual learning space):
  ➢ PreViser documents.
  ➢ seminars on CRSA with associated reading list.
  ➢ Narrated powerpoint presentations on the use of PreViser.
• Seminar: 1 h on PreViser and behaviour change.
  ➢ Recorded and posted as additional resource.

We specifically analysed the data from cohorts summarised in Table 1 below.

To establish the impact of the PreViser pilot (which includes the training for and the actual use of PreViser in the pilot) on our students' behaviour, perception, and knowledge about CRSA, we compared the questionnaire responses of the following pairs of cohorts:

• Group A with Group C.
• Group B with Group D.
• Group D with Group C.

We sent out a call via email for students in the 4th year of the programme (BDS4 – 22 students, *n* = 150) and their care planning clinic teachers (*n* = 10) to take part in our research project on assessing students' behaviour, perception, and knowledge in CRSA, which included two phases in the research project:

Phase 1: Quantitative phase consisting of anonymous questionnaire completion.

After which, we invited those who had completed Phase 1 to attend Phase 2.

Phase 2: Qualitative phase consisting of one-on-one interviews for students and a focus group on MS teams for the teacher group.

### 2.2 Quantitative research

Independent of this study, questionnaires submitted to clinically active students between 2017 and 2021 assessed their behaviour, perception, and knowledge on CRSA (9–16).

Prior to the start of the trial and after completion, the student and teacher participants completed a student or clinical teacher questionnaire, respectively:

• Students were asked questions to gauge their behaviour, perception, and knowledge in terms of oral health assessment.
• Clinical teachers were asked questions to gauge their students' behaviour and perception in relation to caries susceptibility assessment.

Kirkpatrick's four levels of training evaluation framework served as the foundation for the student questionnaire design (Supplementary Appendix 1), which was comprised of four sections:

• Demographic section: four questions covering undergraduate team allocation, sex, age, and year group, as well as if BDS degree is their first degree or not.
• Behaviour section: 13 questions assessing student behaviour towards caries risk assessment.
• Perception section: 13 corresponding questions assessing student perception towards caries risk assessment.

The teacher questionnaire (Supplementary Appendix 2) with its seven questions was designed to complement the student questionnaire by assessing the teacher's perception of the students behaviour/perception and knowledge on CRSA.

### 2.3 Qualitative research

The questionnaire responses were supplemented with online Microsoft Teams student interviews and teacher focus groups post-intervention, using interview guides mirroring the student and teacher questionnaires (Supplementary Appendices 3 and 4, respectively). The purpose was to further explore, in detail, the participant responses for each of the study outcomes along the Affordability, Practicability, Effectiveness, Acceptability, Side-effects, Equity (APEASE) criteria to evaluate behavioural interventions in terms of process (17).

For this phase, we proceeded with purposive sampling within the participants of the quantitative phase for both the student and teacher groups (i.e., from those who completed the survey). A total of five Year 4 students and seven clinical teachers attended the second phase.

### 2.4 Data analysis

#### 2.4.1 Quantitative analysis

This literature review highlighted that the most common method was the use of a questionnaire survey to gather opinions regarding caries risk assessment from students and staff and to also assess the accuracy of their knowledge of caries risk assessment and subsequent management (11–16).

Both questionnaires used variations of a Likert scale, which allowed us to convert the data to an ordinal scale of 1–5. These data were then entered into SPSS for analysis [IBM Corp. released 2021. IBM SPSS Statistics for Windows, version 28.0.1.1 (15) Armonk, NY: IBM Corp].

Due to the variability of our Care Planning rotation in the curriculum, we are looking at data from a cohort-specific perspective rather than focusing on participant-specific data (i.e., 22 T1 and 22 T2 participants were not the same individuals but from the same cohort with the same exposure to training, curriculum, and PreViser in care planning clinics).

As baseline, we also used the data from the same questionnaire that was administered prior to the COVID-19 pandemic in the academic year 2019–2020 as part of an undergraduate research project at FoDOCS, with relevant ethical clearance and consent obtained from the participants.

#### 2.4.2 Qualitative analysis

The sessions were held online using Microsoft Teams meetings. The meetings were run using the guides attached in the Appendices. The sessions were recorded, with the existing written consent from the participants and verbally re-confirmed prior to starting the recording.

The recorded sessions were stored in the Microsoft 360 King's College London (KCL) One Drive (General Data Protection Regulation GDPR compliant) and accessible only to the five KCL members of the PreViser research team (MN, KA, AV, AC, ZM). MS Stream generates automated transcriptions which were then reviewed by two of the team members after the calibration session (AC, MN). MN proceeded with the familiarisation with the transcripts, followed by an initial coding highlighting phrases or sentences—and coming up with shorthand labels or ‘codes’ to describe their content. Next, we identified patterns among the codes, and proceeded with finalising the relevant themes. We returned to the transcripts and reviewed the themes (AC, LC, ZM) before proceeding with the final coding using the agreed themes.

Thematic analysis was the technique employed to identify commonalities and differences in the ideas and phrases that students and teachers articulated in their narratives and that can indicate some degree of importance allocated to a specific thought or occurrence. This research used three aspects of identifying the themes (18):

• Recurrence criterion refers to concepts that are repeated using similar words or phrases.
• Repetition criterion means that an idea is conveyed with the use of the same words.
• Forcefulness refers to the emphasis applied to a concept.

The write-up of the results is presented below.

## 3 Results

### 3.1 Demographic data

Teacher demographics were not recorded. All clinical teachers have at least 10 years of experience in general dental practice and supervise Year 4 undergraduate students 1 or 2 days a week. We provide induction and regular calibration sessions to support them in delivering the curriculum to our students. These clinical teachers (T1 *n* = 11, T2 *n* = 9) supervise students during patient treatment and therefore care planning. The student demographic data is presented in the Table 2 below and shows a similar distribution in terms of age, sex and ICC team in all groups.

**Table 2:** The demographic data of the student participants.

		Age					Sex				ICC Teams				
		20–25	25–30	30–35	Missing	Total (*n*)	Female	Male	Missing	Total (*n*)	21	25	26	Missing	Total (*n*)
Year Group	Group D	18	5			23	13	10		23	6	9	8		23
	Group C	19	3			22	17	5		22	8	8	6		22
	Group B	14	9	1		24	12	11	1	23	7	7	10		24
	Group A	18	1	1	1	21	16	4	1	21	5	7	5	4	21

### 3.2 Quantitative results

#### 3.2.1 Student group

Tests were conducted to determine the association between the categorical data, response, and the BDS4/BDS3 group using Fisher's exact test since the expected cell value was less than 5 for all the questionnaire questions.

The 5-point Likert scales were converted into numbers (strongly agree = 1, agree = 2), and it is important to note that some questions had reverse scales. Since the data were non-normal ordinal data, we conducted Mann–Whitney *U* tests to assess the difference in responses to our CRSA questionnaire from Group A/Group B/Group C, and Group D. The level of significance was set at 5%.

##### 3.2.1.1 Group A/Group C comparison

When comparing the data set for the Group A and Group C cohorts, we find statistically different relevant data with (*p* < 0.05) for the questions in the Table 3 below:

**Table 3:** BDS3-20T2-BDS4-22T1 (Group A/Group C) analysis test statistics.

Question	Mann–Whitney *U*	*p*-value
I carry out a diet analysis for my patients	99.5	<.001
I ask patients about their fluoride use	123	0.003
Over the past semester, I did not perform formal caries risk assessment because of time constraints.	99	<.001
When have you considered fluoride varnish application for High Caries Risk	112	0.007
When have you considered fluoride varnish application for Mod Caries Risk	121.5	0.017
I am confident in using the following Caries Risk Assessment tools: PreViser	100	0.002
CRA includes assessment of the following factors: tick all those that apply:	90	0.006

Looking at the Boxplots.

**I carry out a diet analysis for my patients.**

**Figure 1:**
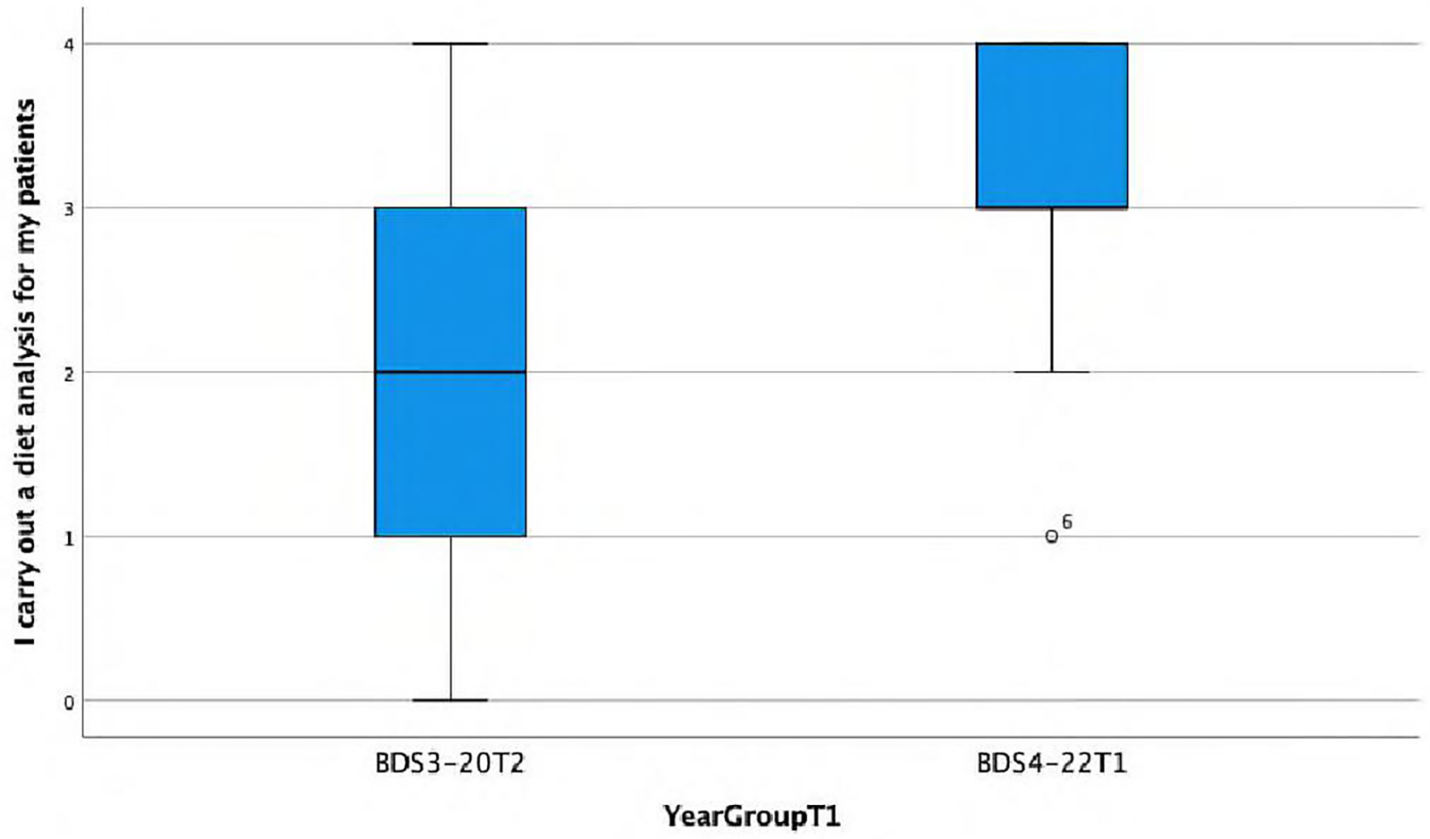
Boxplot showing differences between Group A and Group C in carrying out diet analysis for patients.

There is a statistically significant association observed between the response and the year group (Fisher's exact test, *p* < 0.001), wherein Group C demonstrates a higher frequency (almost always or always) of conducting diet analyses. This is also reflected in the lower median score of 2 and interquartile range (IQR) = 2 in Group A compared with the median score of 3 (IQR = 1) in Group C (Figure 1).

**I ask patients about their fluoride use.**

There is a statistically significant association observed between the response and the year group (Fisher's exact test, *p* = 0.003), indicating that Group C has a higher frequency (almost always or always) of asking patients about fluoride use. This is also reflected in the lower median score of 3 (IQR = 2) in Group A compared with the median score of 4 (IQR = 0) in Group C.

**Over the past semester, I did not perform formal caries risk assessment because of time constraints.**

**Figure 2:**
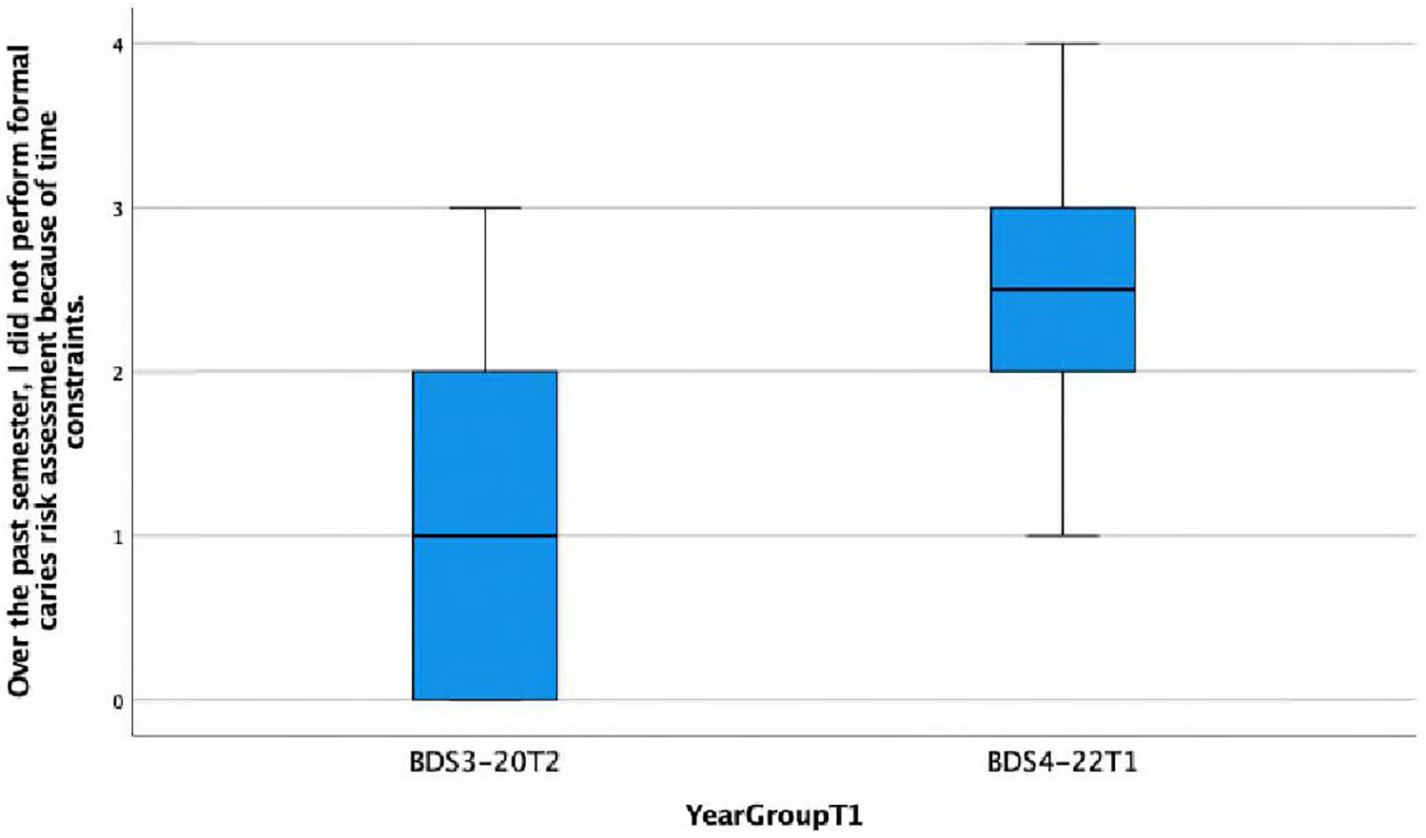
Boxplot showing differences between group A and Group C in performing formal caries risk assessment due to time constraints.

There is a statistically significant association observed between the response and the year group (Fisher's exact test, *p* < 0.001), suggesting that the BDS4 group has a higher frequency (disagreeing or strongly disagreeing) of the statement ‘over the past semester…’ This is also reflected in the lower median score of 2 (IQR = 2) in Group A compared with the median score of 2.5 (IQR = 1) in Group C (Figure 2).

**When have you considered fluoride varnish application for High Caries Risk.**

**Figure 3:**
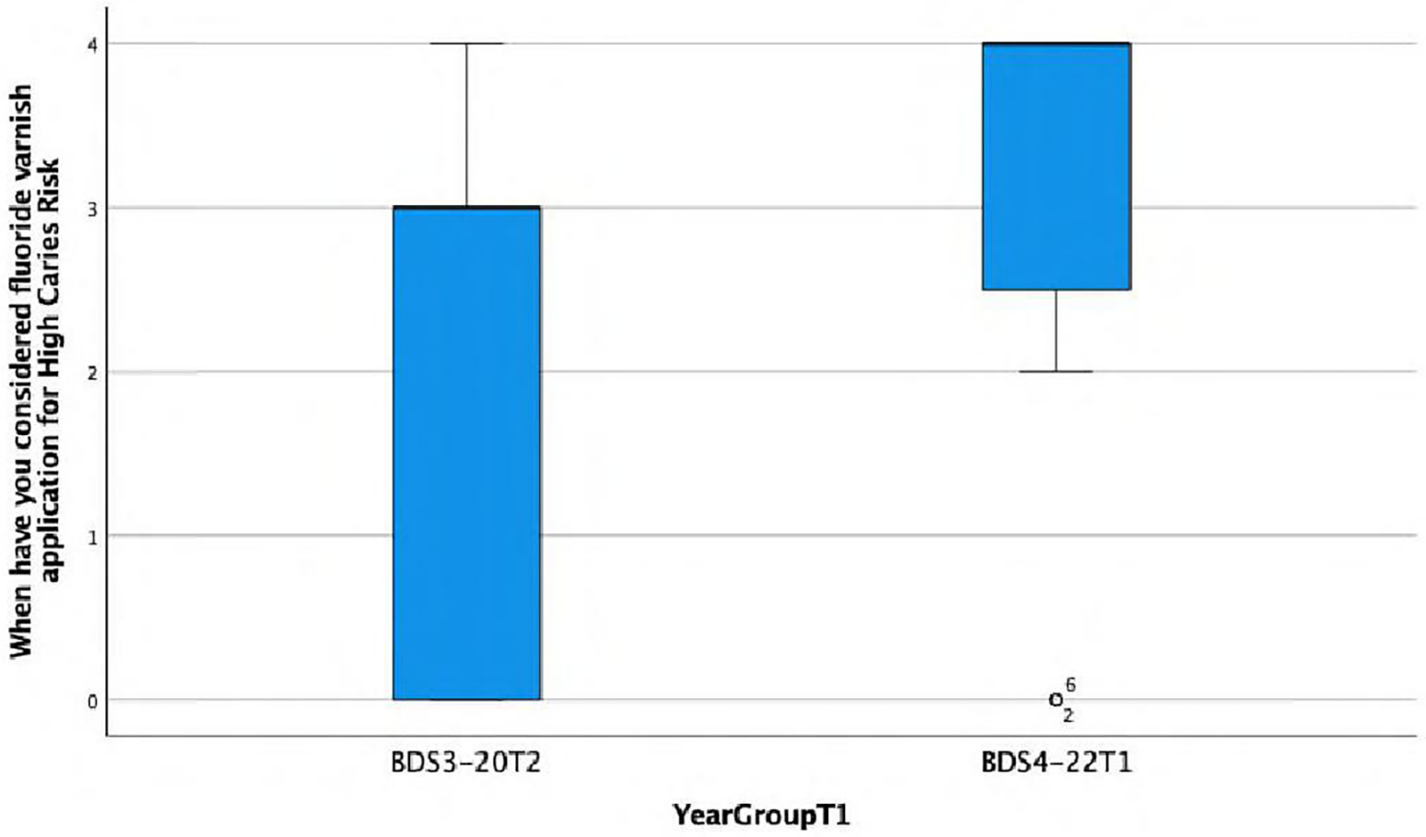
Boxplot showing differences between Group A and Group C in considering fluoride varnish application for high caries risk.

There is a statistically significant association observed between the response and the year group (Fisher's exact test, *p* = 0.007), with the BDS4 group more frequently (always) considering high risk. This is also reflected in the lower median score of 3 (IQR = 3) in Group A compared with the median score of 4 (IQR = 1.5) in Group C (Figure 3).

**When have you considered fluoride varnish application for Mod Caries Risk.**

**Figure 4:**
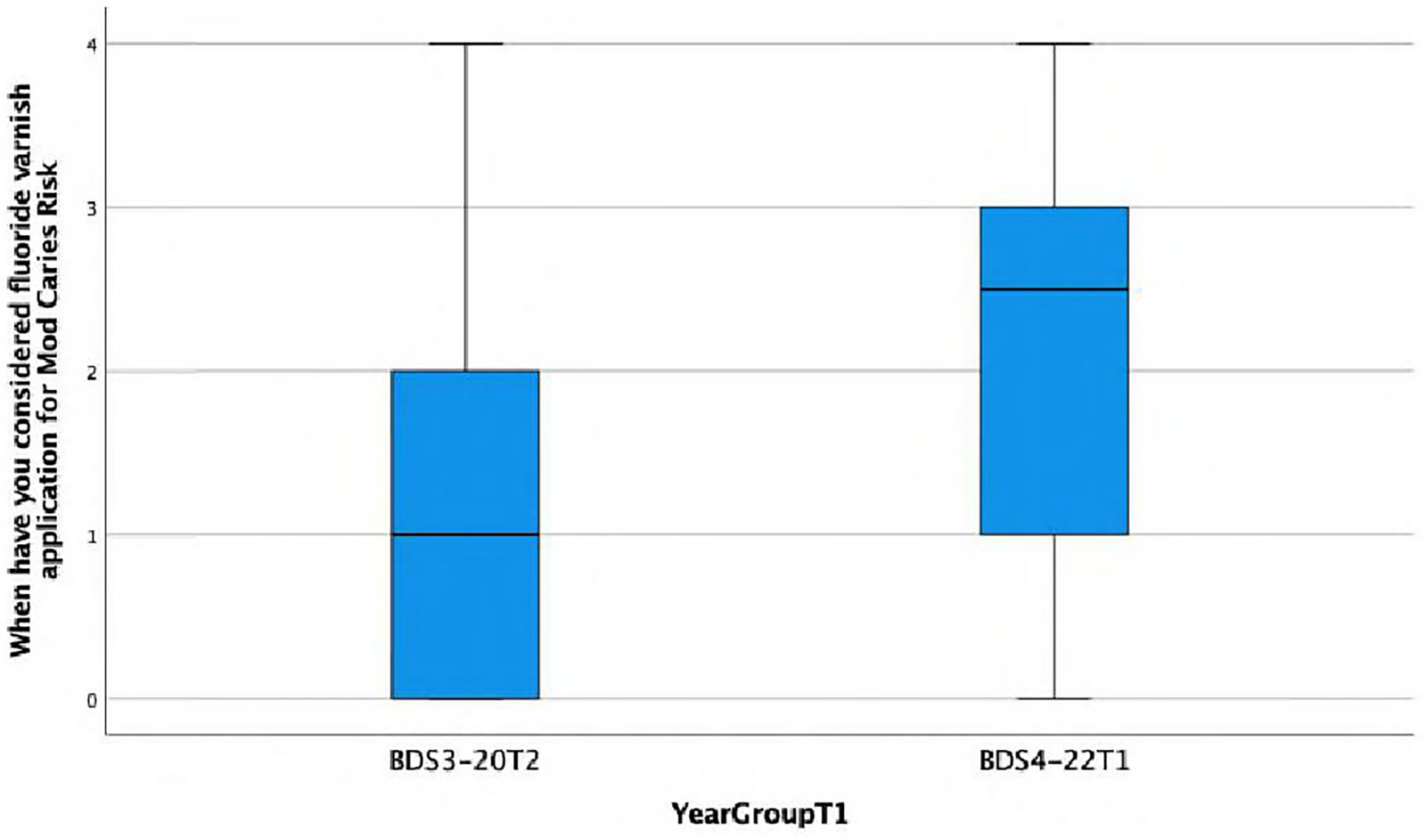
Boxplot showing differences between Group A and Group C in considering fluoride varnish application for moderate caries risk.

There is a statistically significant association found between the response and the year group (Fisher's exact test, *p* = 0.017), with the BDS4 group tending to consider fluoride varnish more frequently (almost always) for patients with moderate caries risk. This is also reflected in the lower median score of 1 (IQR = 2) in Group A compared with the median score of 2.5 (IQR = 2) in Group C (Figure 4).

**I am confident in using the following Caries Risk Assessment tools: PreViser.**

**Figure 5:**
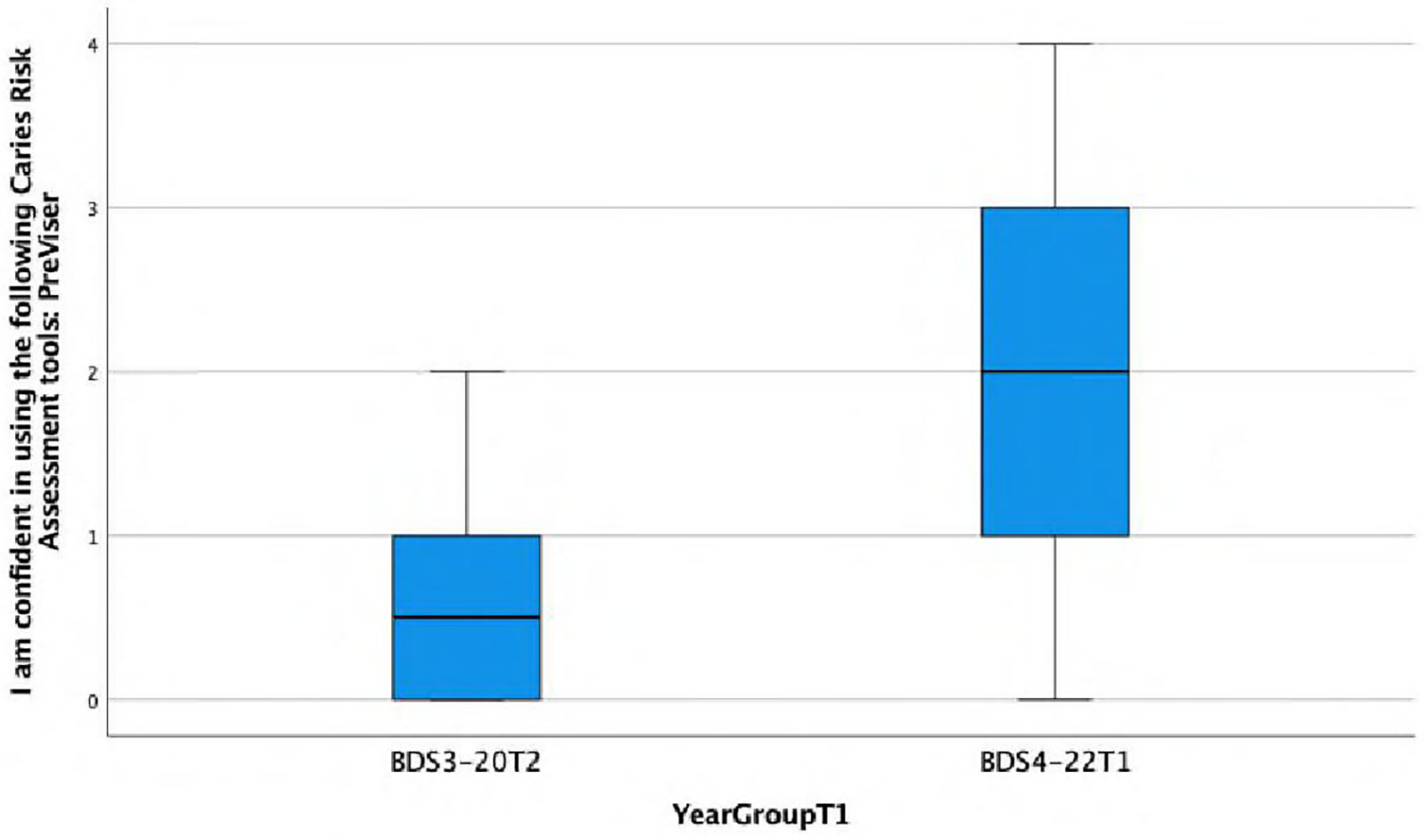
Boxplot showing differences between Group A and Group C in confidence in using a caries risk assessment tools: PreViser.

There is a statistically significant association observed between the response and the year group (Fisher's exact test, *p* = 0.002), with the BDS4 group showing a higher frequency of agreeing or strongly agreeing with the statement ‘I am confident in using …’ This is also reflected in the lower median score of 0.5 (IQR = 1) in Group A compared with the median score of 2 (IQR = 2) in Group C (Figure 5).

**CRSA includes assessment of the following factors: tick all those that apply**

There is a statistically significant association observed between the response and the year group (Fisher's exact test, *p* = 0.006), with the BDS4 group recognising more of the CRSA factors than the Group A. This is also reflected in the lower median score of 7 in Group A compared with the median score of 8 in Group C.

##### 3.2.1.2 Group B/Group D comparison

When comparing the data set for the Group B and Group D cohorts, we find statistically different relevant data with (*p* < 0.05) for the questions in the Table 4 below:

**Table 4:** BDS4-20T2-BDS4-22T2 (Group B/ Group D) analysis test statistics.

Question	Mann–Whitney *U*	*p*-value
I am confident in using the following Caries Risk Assessment tools: PreViser	166.5	0.015

The corresponding Boxplot are as follows:

**I am confident in using the following Caries Risk Assessment tool: PreViser.**

**Figure 6:**
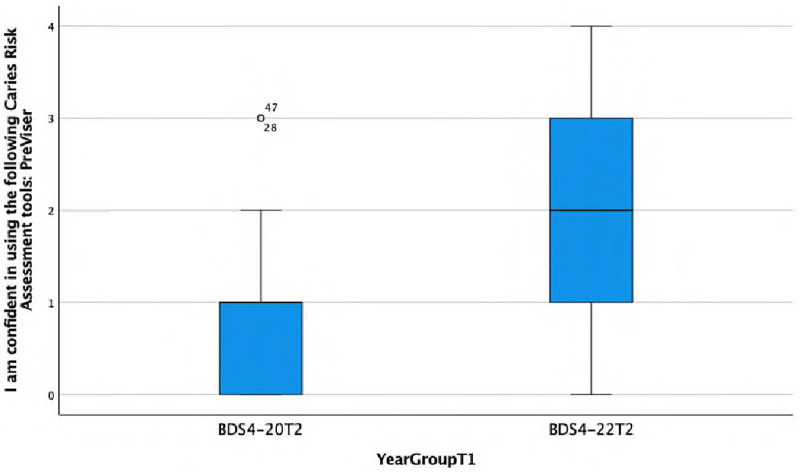
Boxplot showing differences between Group B and Group D in confidence in using a caries risk assessment tools: PreViser.

There is a statistically significant association observed between the response and the year group (Fisher's exact test, *p* = 0.015), with the Group D more frequently disagreeing or strongly disagreeing with the statement ‘I am confident in using …’ This is also reflected in the lower median score of 1 (IQR = 1) in Group B compared with the median score of 2 (IQR = 2) in Group D (Figure 6).

##### 3.2.1.3 Group C/Group D comparison

When comparing the data set for the Group C and Group D cohorts, we find statistically significant differences (*p* < 0.05) for the questions in the Table 5:

**Table 5:** BDS4-22T1/BDS4-22T2 (Group C/Group D) analysis test statistics.

Questions	Mann–Whitney *U*	*p*-value
I carry out a diet analysis for my patients	158	0.039
Over the past semester, I did not perform formal caries risk assessment because of time constraints.	157	0.023

The Boxplots are as follows:

**I carry out a diet analysis for my patients.**

**Figure 7:**
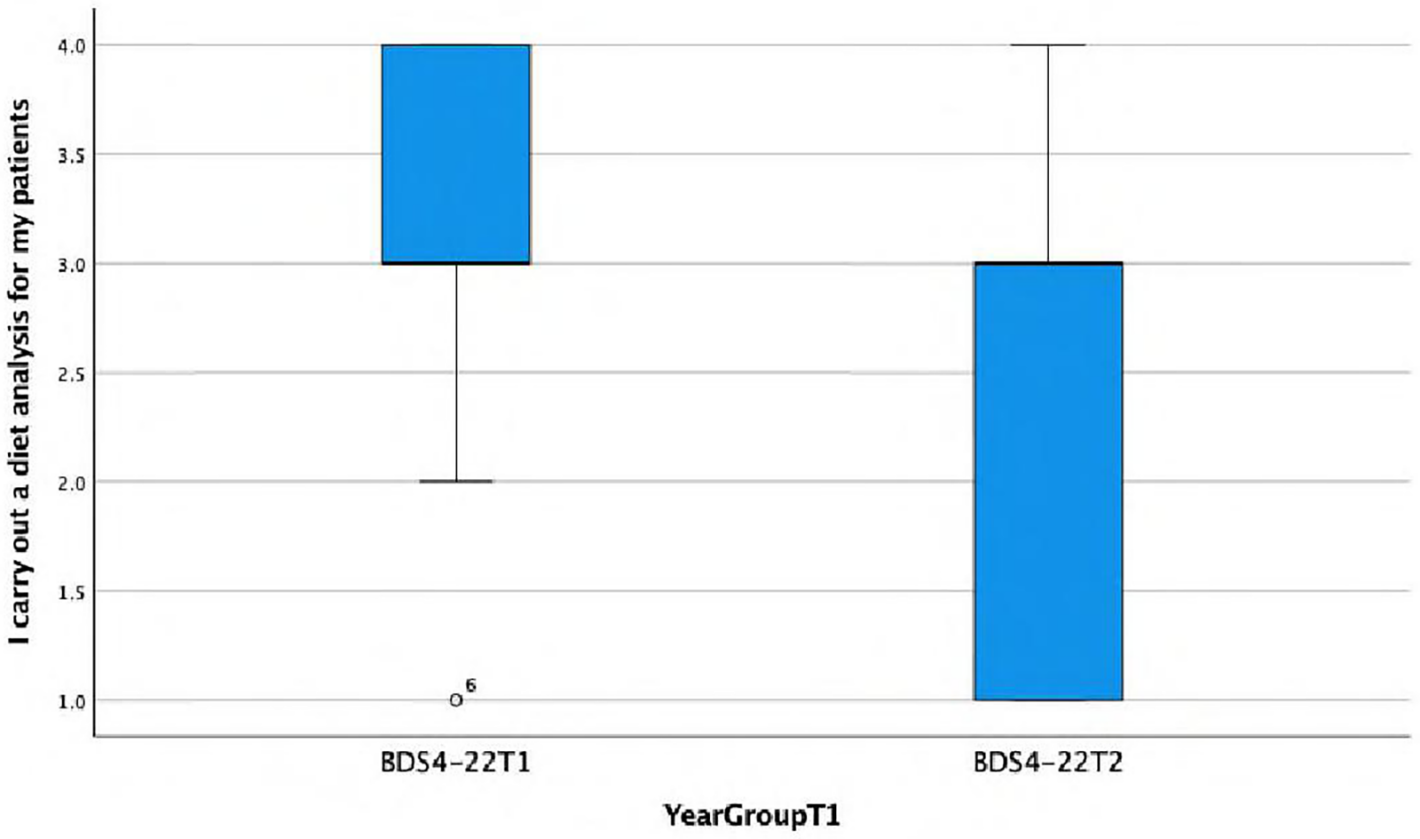
Boxplot showing differences between Group C and Group D in carrying out diet analysis for patients.

There is a statistically significant association found between the response and the year group (Fisher's exact test, *p* = 0.039), with the BDS4-22T1 group more frequently (almost always or always) carrying out diet analysis. This is reflected in a median value of 3, a first quartile (Q1) value of 3, and a third quartile (Q3) value of 4 for Group C, i.e., 50% of the data are above a score of 3. However, Group D while having a median score of 3 has a Q1 value of 1 and a Q3 value of 3, i.e., 50% of the data are below a score of 3 (Figure 7).

**Over the past semester, I did not perform formal caries risk assessment because of time constraints.**

**Figure 8:**
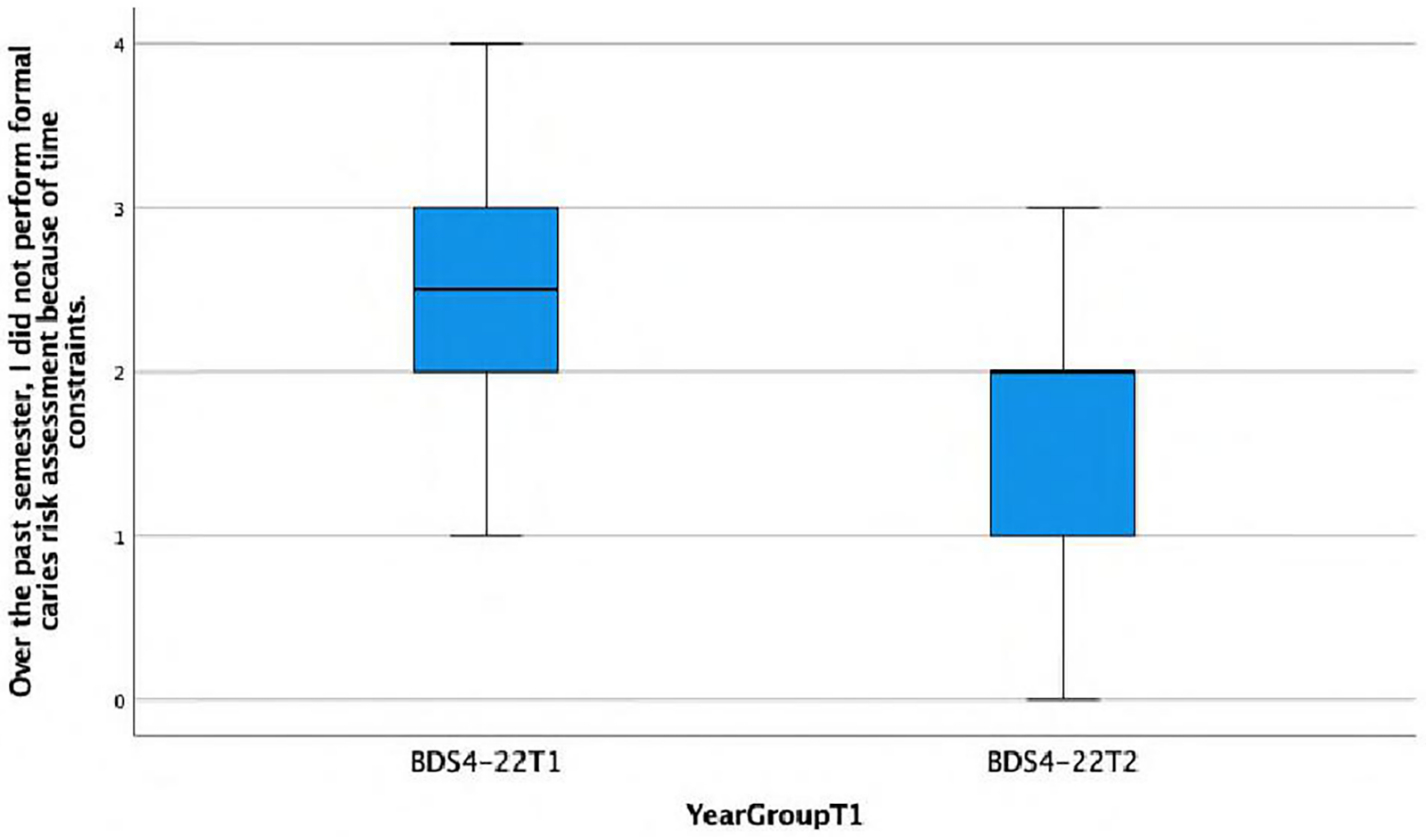
Boxplot showing differences between Group C and Group D in performing formal caries risk assessment due to time constraints.

There is a statistically significant association found between the response and the year group (Fisher's exact test, *p* = 0.023), indicating that Group D's behaviour towards performing a CRSA is more likely to be negatively impacted by time constraints. This is also reflected in the lower median score of 2 (IQR = 1) in Group D compared with the median score of 2.5 (IQR = 1) in Group C (Figure 8).

**I am confident in using the following Caries Risk Assessment tools: PreViser.**

**Figure 9:**
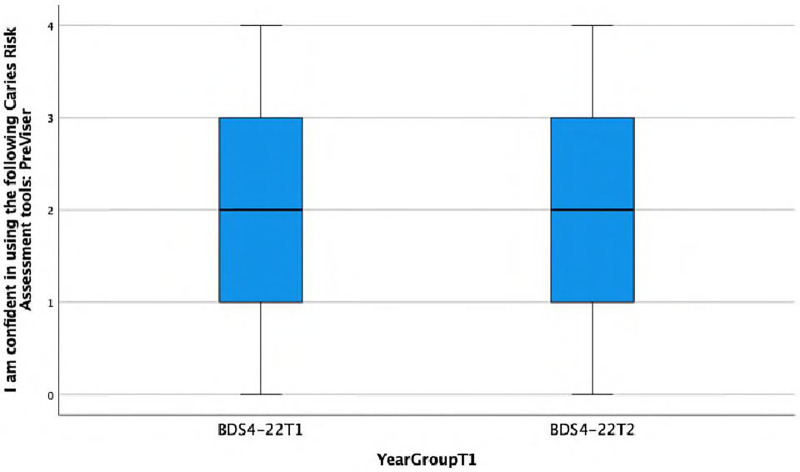
Boxplot showing differences between Group C and Group D in confidence in using a caries risk assessment tools: PreViser.

There was no statistically significant difference observed between the response and the year group, indeed regarding confidence in using PreViser for CRA, both Group C and Group D cohorts provided exactly the same answers, exhibiting the same distribution and median (Figure 9).

#### 3.2.3 Teacher

Regarding the Teacher Data T1 and T2 teachers

Due to rotation variability, we are looking at the data from a cohort-specific perspective rather than a participant-specific data.

The TeacherT1(*n* = 11) and TeacherT2 (*n* = 9) participants belonged to the same cohort and had the same level of exposure to training, curriculum, PreViser in CPC, and student supervision. There was no statistically significant difference observed in the responses provided by the teachers at T1 and T2.

Upon examining the mean plots graph below, it is evident that the teachers usually perceived the following in the Group D cohort compared with the Group C cohort:

• more knowledgeable about CRA.
• more competent about CRA.
• more confident about CRA.
• easier to teach CRA.
• easier to supervise delivering CRA.

### 3.3 Qualitative Resutls

A thematic analysis was conducted, and Table 6 displays the themes together with relevant quotes from the students and teachers.

**Table 6:** Thematic analysis results summary.

Themes	Student quotes	Teacher quotes
PreViser Impact	Student 2: I think that definitely, my knowledge over this year has increased a lot in terms of caries risk assessments. Especially, I think you know, PreViser has been a big part of it because there was a couple video and things that we needed to watch, and the training we needed to do as well for the PreViser, which definitely helped.	Teacher 7: Do you use it in practice? And I said, well, I don't. But I do think it's a very useful tool. Especially for inexperienced dentists, so newly qualified dentists, students when they can't really work out the risk assessment, can't really work out the risks very easily. I think it's really useful.
Competence	Student 5: My competence kind of based on when the clinician comes up afterwards and does the same thing and see if they add anything extra to my kind of history and examination and I would say that: The one time I maybe, maybe my competence is being challenged is around older restorations, where there's leaking margins—that sensitivity I don't think is quite there. But I think otherwise picking up disease seems to be equivalent to what the clinician finds.	Teacher 4: The more we’re able to use it and practice with it, I think, the more competent we will get.
Confidence	Student 1: I think I would be able to carry out an oral health risk assessment now by myself. I think I've had enough practice now and enough knowledge to be able to put it into practice confidently.	Teacher 6: It is very new and still we are in private practice; we are not using this system. So just we should give, you should give it time. PreViser also at the beginning we said that ‘no, why, why’ but after a short time, I'm sure that we will find how advantageous it is, how good it is.
Education/Pedagogy	Student 5: I think I've got a fair grounding and understanding of what other major risk factors for developing both of those diseases, including the general risk factors for patients and then also maybe those little extra ones that might increase their susceptibility.	Teacher 1: With the case with risk assessment it's just how we were taught, the dental students So I have this knowledge bank already in my brain. So I have to rely on my training and expertise in oral healthcare assessment, which I think is competent.
Communication (including validation/validity)	Student 2: And I think also to assess their interests. And you know, because after you do all health assessment you have the discussion with them. But like if they're for example, someone that's not really motivated by like, you know, like we've had lectures on motivational interviewing and how to, you know, ask, advise, act and things like that.	Teacher 3: It's something, really, something there for the patients to see and they understand it more in, you know, in layman terms. Which is eventually, you know, is all about treating them and getting them to change their ways and diet and risk and things like that. They understand these things in a more layman fashion. Whereas with the students at that stage of their career or education, they're still trying to learn the skills of communication. How to communicate in a way which is not so technical.
Specific Training	Student 2: And I also think like having kind of an in person training session would be very, very beneficial to us using PreViser.	Teacher 3: There needs to be quite a meticulous training programme so that people are quite efficient with it. I think that you know, in terms of the tool itself is brilliant. I think the issue generally on clinic is time and so if there's an efficient training programme and maybe like a day is not only a training programme for the students for the staff as well.
Role modelling	Student 1: It depends on the tutor … and I think because of that first session then afterwards no one did it just because they thought oh this tutor doesn't want us to do it, so there's no point in doing it.	Teacher 1: They're keen to know what I do in practice. And I think one of them did say, are you using this Doctor… and I said ‘No, I'm not using it in practice’. And perhaps, I don't know, then there could be a downside for me telling them that. Because then it probably makes them think why are they doing it.
Embed in Electronic Patient Record	Student 3: The only thing is, I think if it was integrated, would say salud or if not just like one system we used to access both like if it was integrated into the history, for example, that would have been really good because I think having multiple programmes to use it has it does make it a little bit difficult	Teacher 2: The less obstacles to them being able to do it—you know, the kind of logging in or you know, all that sort of stuff—the more streamlined it is, the easier it is for them to not have an excuse not to do it.
Repetition/Time constraints	Student 5: Sometimes if there are time pressures on the clinic, especially getting into UM diagnostics, like radiology or there's a lot waiting Maybe the detail, the depth of the oral health risk assessment that you go into. And also the integration, I mean using multiple systems is never um, that's always tricky, isn't it?	Teacher 1: I think my initial concern was that it would take them away from the learning of clinical practice, but having seen the few students who did it, it didn't seem to have an impact on their time with the patient, and because they didn't ask me for any involvement or engagement, I can't see how it's going to take up my time in addition.
Systematic/Approach	Student 3: I think having PreViser is good, UM in like sort of, you're having that systematic approach like you said. It takes you through the whole process and you kind of can discuss the reasoning for different questions. You're asking the patient as you're going through.	Teacher 3: For learners I think it's really good, especially in terms of grasping (the whole the in terms of grasping) treatment planning itself, in terms of the different aspects of treatment planning. You know the order in which you treat the patient and how you're going to, you know, work on the basics first before you go to the definitives.
Specific use (including audit, triage, QAQE, indication)	Student 4: I think also just initially when you’re starting clinic, it would probably be good to do it for all patients just so that you can understand how to do it.	Teacher 4: It'll be more case of doing it at initial visit when they're through with that consultation. When they come back, they can do PreViser again. I don’t think it's something we can do at every patient visits.
More Experience using it	Student 1: So I think PreViser has needed, I find that I prefer using PreViser than not using PreViser because it does make it easier but then but then again, I've only used it about four times but I haven't really been able to because I haven't really had the opportunity.	Teacher 2: I've only ever done it when I've been supervising students. And obviously it's like anything, the more they do it, the quicker they'll be at doing it
Ease of Use (including independent use)	Student 2: I actually think it's been a very like straightforward, and I think it's been done in a really well like stepwise manner.	Teacher 2: I think as long as the students knew how to log in and kind of do all that, the technical stuff it was. It was quite fine. If they didn’t have their or, you know, they weren’t familiar with how to log in and do that sort of thing, then it could be a bit of an issue because then they would have to spend time trying to figure out how to do that and then that take up time
Patient care/Practice Setting	No student comments	Teacher 7: at Guy's is that you don’t always get that follow up and that sort of continuing treatment and the recalls like you would in practice. I think it'd be a lot easier in practice to do it then it would be in hospital.

#### 3.3.1 Student group

Overall, there was a positive perception from the students towards PreViser, highlighting that PreViser is straightforward to use.

They liked the systematic approach it gave to oral health risk assessment. Having this clear structure translated into good communication with the patients as it highlighted the causes of disease and prompted topics of discussion. It can enable difficult conversations with patients as there is ‘evidence’ to back up the clinical recommendations.

The students generally seemed confident in oral health risk assessment and felt they could complete this competently and independently. This reflected their training throughout the Undergraduate degree programme, in addition to PreViser. However unfortunately, many students felt they had not had enough exposure to PreViser on clinic. This was partly due to the infrequency of care planning clinics, where PreViser was being used, and also due to a lack of motivation to use the programme by both students and teachers. Some mentioned forgetting to use the programme, or due to time pressures, and the majority of students commented on the significant influence of the teacher's preferences on whether, and to what extent, oral health risk assessment was performed.

The tool itself can be viewed as repetitive if the students ask questions in addition to those they are instructed to ask regularly. Embedding PreViser into existing electronic patient record systems would support its use, as would more training and small gaps between training and opportunity for clinic use.

It can also be good for triaging patients especially in a large hospital. The students also mentioned the possible use of the tool for auditing patient records in terms of oral health assessment and when looking at resource allocation (treatment).

#### 3.3.2 Teacher group

Several teachers owed their low self-reported confidence and competence in using the PreViser software to the lack of familiarity and limited experience in using the tool. This may explain the ‘hands-off approach’ when supervising students using PreViser in the clinic. Further calibrated training and guidance was deemed necessary with many reporting the need for additional and more frequent opportunities to practice using the software. Teachers feel confident in their own knowledge, training, and experience to complete oral health assessment independently. However, they were able to recognise the benefit of PreViser as an educational tool for dental students and young dentists to help establish sound foundations, as well as to clinically facilitate communication with patients and support behaviour change.

The teachers had not used PreViser consistently yet felt able to comment on it on the basis that students seemed to be getting on well with it. They recognised the strong influence they have on students' behaviours and acknowledged the need to better encourage students on the benefits and uses of risk assessment.

The following are the teachers’ comments on the opportunities related to PreViser:

➢ It is good for education purposes.
➢ Students could use it with their patients with instructions on a laminated form.
➢ It is good for continuity of care in practice.
➢ Patients engaged with it more than usual.
➢ It improves communication with patients:
  • chairside as helps speaking to patients about their oral health in more layman's terms
  • take home information covered

The teachers also highlighted what they perceived the following to be barriers to using PreViser:

➢ lack of time and burden for patient
➢ did not feel it compromised clinical practice not to use this tool although an oral health risk assessment is required
➢ current dental contracts do not allow a place for it in practice
➢ need a meticulous training programme and has to be for all patients in all clinics
➢ cannot be used at every visit, perhaps look specifically to initial and recall visits

## 4 Discussion

Computerised tools incorporating validated algorithms and/or the latest evidence base provide consistent and reproducible assessment of risk to support clinical judgement. There are two systems, PreViser and the PRA (Periodontal Risk Assessment), that have been validated in longitudinal trials for assessing the risk of periodontal disease. Multiple systems (e.g., CAMBRA, Cariogram) have been established for caries risk assessment, although no predictive algorithm has been validated (12, 13, 19). Similarly, there is good knowledge of the risk factors for tooth wear or oral cancer, although no algorithm that combines these factors has been shown in clinical trials to predict disease accurately (20–22). It would, however, be wrong to take this as a reason not to assess the risk and simply focus on fixing the disease. According to WHO, ‘Estimation of the potential impact of a health hazard can never wait until perfect data are available since that is unlikely to occur’ and ‘Considerable gains can be achieved by reducing the risks of factors that are already known’.

PreViser as previously mentioned was chosen as it supports a philosophy of tailored person-focused care based on risk/susceptibility assessment, in line with the pedagogy developed in the undergraduate curriculum. Teaching the new generation to embrace preventative approach will hopefully bring change to the treatment-focused care plan approach in general dental practices.

The impact of introducing PreViser to the 2022 Year 4 cohort was gauged in comparison with preceding 2020 cohorts as described in our Participants section using as base line data from the same questionnaire on Caries Risk/Susceptibility Assessment that was administered prior to the COVID-19 pandemic in academic year 2019–2020 as part of an undergraduate project at FoDOCS with relevant ethical clearance and consent obtained from the participants.

### 4.1 Group A/Group C

Generally, the items that exhibited statistical significance when reviewed show a better behaviour, perception, and knowledge of the Group C cohort in comparison with the BDS3-20T2 cohort, except for their behaviour towards performing a CRSA which was more likely to be negatively impacted by time constraints that they associated to the process.

We can attribute these differences to the impact of the PreViser training and sensibilisation as the COVID-19 pandemic affected both cohorts (end of the Group A cohort studies and end of Year 2 and all of Year 3 for the Group C cohort).

### 4.2 Group B/Group D

We note that the Group D students feel more confident using the PreViser as a CRSA tool. This can be attributed to the impact of the PreViser training, sensibilisation, and use as the COVID-19 pandemic affected both cohorts (end of the Group A cohort studies and end of Year 2 and all of Year 3 for the Group D cohort.

### 4.3 Group C/Group D

When comparing Group C and Group D data, we note that the students from the Group C cohort were more likely to carry out a diet analysis for their patients and were less likely to be negatively impacted by time constraints compared with the Group D students. Both cohorts were equally confident in using PreViser for CRA.

We would perhaps expect clearer differences as Group D also applied PreViser, but the training was more removed from their experience.

The student and teacher interviews provided us more qualitative insight into behaviour, perception, and knowledge on CRSA and the factors impacting them. Generally, we noted the following across the discussions:

Although competence and confidence appear high (knowledge about oral health risk rather than actually being able to do in practice), they acknowledge that they would need more support to use it chairside. The research shows a need to improve students' confidence in performing risk assessment. At the University of Sydney, 60% of third-year students and 71% of fourth-year students found the caries management system useful on clinics. However, 44% of the third-year students found that the protocols are complicated (11). If the students were more comfortable with the protocols, better care could be provided for patients. In Tehran, over 50% of students did not believe that their ability was enough to perform caries risk assessment (13).

The main barrier listed to using PreViser rests in the fact that clinical teachers either prefer their own ways of assessing or do not know how to use the tool and therefore did not encourage using it. The study suggested that perhaps the staff members did not embrace the need for caries management programme despite undergoing training. Staff opinions could have negatively impacted the students' views, thus leading to poor completion of the caries risk assessment forms (23).

Embedding PreViser into existing electronic patient record systems would support its use, as would more training and small gaps between training and opportunity for clinic use. Students' knowledge on risk assessment and appropriate management needs continuous reinforcement and improving. One study reported that only 44.1% of medium and high-risk patients received fluoride varnish. When the patients were reassessed, 25% of patients had been wrongly categorised as medium, when they were in fact high-risk patients (19). Continuous education surrounding caries risk assessment can positively influence its understanding and use. This is also supported by recent evidence from a study (24) at the University of Michigan School of Dentistry. A caries risk assessment model was first introduced in 2011, and soon after its launch, only 43% of patient charts had a completed caries risk assessment. However, from an unspecified 2-year time period close to publication, it was completed in 80%–88% of the cases. The 7-year retrospective study showed that the completion of risk assessment by the dental students had risen over time (25).

This more importantly infers that as a profession, including both students and qualified dentists, on-going and consistent education on caries risk assessment and management needs to occur in order to provide the best patient care in accordance with the current guidelines.

Those who did use PreViser highlighted that it is straightforward to use.

The main positive finding/point is that it is systematic, enables conversations, can alert the gaps between what the student has seen in a person's mouth and what PreViser says about the state of their oral health.

It can enable difficult conversation with patients as there is ‘evidence’ to back up the clinical recommendations.

It can also be good for triaging patients especially in a large hospital.

The students also mentioned the possible use of the tool for auditing patient records in terms of oral health assessment and when looking at resource allocation (treatment).

Our findings also support the fact that seniority in the programme aligns with a better behaviour, perception, and knowledge towards CRSA. A very recent study assessed the opinions of fifth year dental students from 16 different French dental schools. The results showed positive use of caries risk assessment, with 80% using it in clinical practice. However, it highlighted that this does not necessarily translate to correct and appropriate care planning, as only 55.1% implemented preventative regimes according to the designated risk level (14). Confidence among students also increased with years of education, showing a positive association between years of teaching and perceived confidence (15).

The research suggests an underperformance of accurate caries risk assessment by dental students but also in general practice. One study involving general dental practitioners in France showed that an astonishing 38.4% of respondents did not use caries risk assessment as part of their routine care. Only 4.5% of those claiming to perform caries risk assessment used a specific form. The socio-demographic characteristics of the dental practitioners did influence whether or not caries risk assessment was used (16).

## 5 Limitations

### 5.1 COVID-19 impact and PreViser pilot study

We maintained original aims of assessing the benefits of using PreViser in terms of Undergraduate Education and patient care. Considering COVID-19-related constraints in particular to Undergraduate clinics, we had to apply a 12-month delay (started September 2021) for the start of our project, and our care planning clinics ran but with different staff rota each week and students attending on a 1 every 4 weeks rota. Also, it is important to note that only the computers in the care planning clinics were cleared for PreViser use (post Information Governance discussions with the hospital). This limited our staff and students' familiarisation and consistent/systematic use of the PreViser.

### 5.2 Undergraduate clinics at FoDOCS

Our Undergraduate clinics do not have a formal review/recall framework. The usual pathway is discharging of patients back to their GDP after we have finished the course of treatment agreed at care planning. If patient care is long enough to include a review/recall as required by patient oral health risk assessment and preventative planning, then it is carried out for that patient while still under our care. We could not support a longitudinal approach to CRSA, i.e., at baseline and then review at set recall intervals as would be recommended.

### 5.3 Questionnaires

Looking at the Teacher questionnaire, in Question 3, the Extremely confident and Very Confident answer options were reversed in the sequence of answers and points. But since none of the teachers chose either one of the options, the data was not impacted.

## 6 Conclusions

From the student data, the main impact of the PreViser pilot comes from the training set in place in preparation for the use of this CRSA tool in clinic to support our care planning process. The use was not as consistent as it should have been due to specific undergraduate clinic rotations with additional disruption due to COVID-19-related changes and the limitations of PreViser use related to GDPR and NHS trust requirements for patient data safety. The students appreciated its straightforward use, its help triaging patients in terms of their CRSA, and its use in allocating resources (treatments).

From the teacher perspective, the entire cumulative benefit of training and use (even limited) had an impact on our students' knowledge, competence, confidence regarding CRSA and made teaching and helping them deliver CRSA easier, although the importance of CRSA was felt to be more evident right after training.

Both the students and teachers recognise the positive effects of using PreViser as it enables the following:

• a systematic approach to CRSA.
• conversations with patients and supervisors about CRSA.

But that to have full benefit from its use, we have to work on the barriers:

• Time constraints: looking at repetition between tool (PreViser) questions and expected clinical questions.
• Use in all clinical environments, not just care planning clinics.
• Training of all staff, not just those facilitating care planning clinics.
• Training of all clinically active students, not just those involved in care planning clinics.
• Use of laminated cards in all clinical environments.
• Updated/reminders throughout the year.

The traditional ‘drill and fill’ mentality is still sometimes overshadowing the evidence-based minimally invasive protocols. To help prevent this, the dental curriculum from now on must reflect this preferred method of care. There is an opportunity for universities to shift away from treatment quotas, to enable students to focus more on holistic patient-centred care and reflect more on their personal development. More perseverance is needed and further emphasis during education to ensure that the students become confident clinicians in caries risk assessment and carry this into their lives as general dental practitioners.

The oral health curricula of the future must address the lack of knowledge, lack of motivation, and/or lack of confidence in CRSA not just from the students but more importantly from the teachers who should be role-modelling best practice.

We would recommend further research to understand the factors influencing student behaviour, perception, and knowledge in CRSA with the aim to make recommendations on a preferred approach and tool to help streamline CRSA education.

For your information, all abbreviations used in this manuscript are listed in Supplementary Appendix 5.

## Data Availability

The raw data supporting the conclusions of this article will be made available by the authors, without undue reservation.
