# Supplementary material for: An evaluation of the use of caries risk/susceptibility assessment in an undergraduate dental curriculum
Source: Front Oral Health. 2024 Jan 29;4:1290713. doi: 10.3389/froh.2023.1290713 (PMC10859480; doi:10.3389/froh.2023.1290713)
Supplement: Supplementary file 1 [file Datasheet1.docx]

**Appendix 1: Student Questionnaire**

**CRA Program Evaluation Questionnaire**

Dear student,

We would like you to look back and reflect in particular on Caries Risk Assessment education within your Dental Curriculum. We need your help to understand how to improve the CRA education at the Dental Institute. Your input on the education you have received will allow us to improve the CRA education going forward for future generations.

Your participation in this study is voluntary and the information you give us is anonymous and confidential. If you choose to participate, it is very important that you respond to all of the questions and honestly, no one will be able to trace the response back to you and what you say will have no negative impact on you as this is an anonymous and confidential questionnaire.

Carefully read and answer each question. Please choose and mark the response option that best represents your opinion about Caries Risk Assessment.

**Please identify the clinics in which you have practiced by ticking the appropriate box: multiple answers apply**

ICC Fl21 ICC FL25 ICC 26

**Please answer the following questions on your demographic profile?**

Gender: Female Male Prefer not to answer

Age: 20-25 25-30 30-35

35 or more Prefer not to say

Will BDS be your first qualification? Yes No

Do you have any other prior qualification? Yes No

If Yes what is it?______________________________

**Questions**

The following questions assess your attitude toward Caries Risk Assessment as a whole (education and practice).

Looking at the past clinical year and the education you have received on Caries Risk Assessment (CRA):

1. When reviewing patients’ medical history I look up medications to check on how they may impact CRA:

always/almost always/often/sometimes/not at all

1. I carry out a diet analysis for my patients…

always/almost always/often/sometimes/not at all

1. I ask patients about their fluoride use

always/almost always/often/sometimes/not at all

1. I use the modified ICDAS codification to identify caries stage for each patient?

always/almost always/often/sometimes/not at all

1. I perform CRA on each patient I care for?

always/almost always/often/sometimes/not at all

1. Over the past semester, I have performed a CRA for
2. Patients with poor OH

all/most/a few/very few/none at all

1. Patient’s with a high sugar diet

all/most/a few/very few/none at all

1. Patient’s with medical condition impacting their salivary function

all/most/a few/very few/none at all

1. Over the past semester, when carrying out a CRA I used a recognised CRA tool such as
   1. Cariogram

always/almost always/often/sometimes/not at all

- 1. CAMBRA

always/almost always/often/sometimes/not at all

- 1. A different tool, please state which here:_______________

1. Over the past semester, I did not perform formal caries risk assessment because of time constraints.

Strongly agree/agree/neutral/disagree/strongly disagree

1. My risk assessment impacts my patient management and care planning

All the time//almost all the time/sometimes/not very often/not at all

1. When have you considered the following fluoride therapy:
   1. fluoride tooth paste prescription (2800ppm)
      1. High Caries Risk Patients

always/almost always/often/sometimes/not at all

- - 1. Mod Caries Risk Patients

always/almost always/often/sometimes/not at all

- - 1. Low Caries Risk Patients

always/almost always/often/sometimes/not at all

- 1. fluoride varnish application
     1. High Caries Risk Patients

always/almost always/often/sometimes/not at all

- - 1. Mod Caries Risk Patients

always/almost always/often/sometimes/not at all

- - 1. Low Caries Risk Patients

always/almost always/often/sometimes/not at all

1. How often do you review your patient’s caries risk?

always/almost always/often/sometimes/not at all

1. Does your care plan include patient education?

always/almost always/often/sometimes/not at all

1. Have you used CRA as a patient education tool?

always/almost always/often/sometimes/not at all

The following questions assess your practice of Caries Risk Assessment during your clinical year.

Looking at your experience with CRA in the past clinical year answer the following questions:

1. I feel I have an adequate knowledge on identifying the principal caries risk factors

Strongly agree/Agree/undecided/Disagree/Strongly disagree

1. I am not confident in assessing patients diet in regards to their caries risk

Strongly agree/Agree/undecided/Disagree/Strongly disagree

1. I believe it is important for CRA to assess the patient’s fluoride use

Strongly agree/Agree/undecided/Disagree/Strongly disagree

1. I have had sufficient information and training on the ICDAS for caries detection and monitoring.

Strongly agree/Agree/undecided/Disagree/Strongly disagree

1. CRA is essential in the development of all patients’ care plans

Strongly agree/Agree/undecided/Disagree/Strongly disagree

1. I do not think CRA is appropriate for all patients

Strongly agree/Agree/undecided/Disagree/Strongly disagree

1. I have had to consult sources outside the materials I was given in my course, to find out all the information I need to be able to perform CRA on patients

Strongly agree/Agree/undecided/Disagree/Strongly disagree

1. I am confident in using the following Caries Risk Assessment tools.
   1. Cariogram

Strongly agree/Agree/undecided/Disagree/Strongly disagree

- 1. CAMBRA

Strongly agree/Agree/undecided/Disagree/Strongly disagree

- 1. PreViser

Strongly agree/Agree/undecided/Disagree/Strongly disagree

1. My clinical teacher ‘s advice on care planning is consistent with my education on patient risk based management

Strongly agree/Agree/undecided/Disagree/Strongly disagree

1. I am unclear about the non-operative preventive therapy choices available based on the patient’s CRA

Strongly agree/Agree/undecided/Disagree/Strongly disagree

1. I am confident in using CRA to determine my patients’ review/recall timeframe Strongly agree/Agree/undecided/Disagree/Strongly disagree
2. Explaining CRA results to my patient is an area I need more help with

Strongly agree/Agree/undecided/Disagree/Strongly disagree

1. Patient education is useful for patient adherence with preventive recommendations

Strongly agree/Agree/undecided/Disagree/Strongly disagree

The following questions assess your actual knowledge in regards to Caries Risk Assessment.

Using the knowledge you have acquired over your entire undergraduate curriculum up to date, please answer the following questions to the best of your ability:

1. CRA includes assessment of the following factors:
   1. Plaque control
   2. Frequency of carbohydrate intake
   3. Quality of carbohydrate intake
   4. Fluoride
   5. Saliva quality and quantity
   6. Caries incidence
   7. Caries prevalence
   8. a,b,d,e,f
   9. all of the above
2. A patient’s medical condition and medications they may be taking to treat these can affect the caries risk.

true/false/DNK

1. The following are appropriate Diet analysis methods
   1. Take home diet questionnaire given to the patient to bring back next appointment
   2. Chair side review of diet questionnaire with the patient recalling general habits
   3. 24 hours Dietary recall analysis done chairside with the patient
   4. a and c
2. Which of the following is an adjunctive caries detection tool
3. caries detector dyes
4. Laser fluorescence
5. Fiber Optic Transillumination
6. Radiographs
7. All of the above
8. Identify the category(ies)of patient below requiring a CRA
   1. Patients with a high caries index only
   2. Patients with poor OH
   3. Patients with a high sugar diet
   4. Patient’s with medical condition impacting their salivary function
   5. All of the above
9. What is a personalised care plan?
10. Care plan identified based on patient’s preferences
11. Ideal Care plan identified for all patients
12. Care plan identified based on the patient’s specific oral health risks (including CRA)
13. Care plan for which the patient has consented
14. All of the above
15. Which of the following tools can be used for CRA?
    1. Cariogram
    2. PreViser
    3. CAMBRA questionnaire
    4. All of the above
16. An individual with a past history of at least 1 new carious lesion within the past year is at high risk for caries true/false/DNK
17. Fluoride varnish should be prescribed for:
    1. All patients
    2. Moderate and High Caries risk patients
    3. High Caries risk patients
18. High caries risk patients should have bitewing radio­graphs
    1. every 6 months
    2. every 3 months
    3. every 12 months
    4. every 24 months

1. What is the review timeframe for the following patients:
   1. Low caries risk
      1. every 3 months
      2. every 6 months
      3. every 12 months
      4. every 24 months
   2. High Caries Risk
      1. every 3 months
      2. every 6 months
      3. every 12 months
      4. every 24 months___________________
2. The following items are identified as part of patient education?
   1. Diet analysis and counselling
   2. OHI
   3. Fluoride use
   4. Salivary pH and flow determination
   5. Remineralisation strategy
   6. All of the above
3. Which of the following is dependent on patient education?
   1. Preventive measure compliance
   2. Patient consent for treatment
   3. Review/Recall protocol compliance
   4. Success of overall care plan
   5. Caries Disease Process
   6. All of the above

You have reached the end of this survey. We would like to thank you again for your genuine and essential participation and the time and care you have put in.
