# Supplementary material for: An evaluation of the use of caries risk/susceptibility assessment in an undergraduate dental curriculum
Source: Front Oral Health. 2024 Jan 29;4:1290713. doi: 10.3389/froh.2023.1290713 (PMC10859480; doi:10.3389/froh.2023.1290713)
Supplement: Supplementary file 2 [file Datasheet2.docx]

Appendix 2: Teacher Questionnaire

CRA_Teachers' survey

Start of Block: Default Question Block

Q1 Thinking about their knowledge of Caries Risk Assessment (CRA), I think this cohort is...

- Extremely knowledgeable (1)
- Very knowledgeable (2)
- Moderately knowledgeable (3)
- Slightly knowledgeable (4)
- Not at all knowledgeable (5)

Q2 Thinking about their carrying out CRA with patients, I think this cohort is...

- Extremely competent (1)
- Moderately competent (2)
- Slightly competent (3)
- Neither competent nor incompetent (4)
- Slightly incompetent (5)
- Moderately incompetent (6)
- Extremely incompetent (7)

Q4 Thinking about their carrying out CRA with patients, I think this this cohort is...

- Not at all confident (1)
- Slightly confident (2)
- Moderately confident (3)
- Extremely confident (4)
- Very confident (5)

Q3 Thinking about the importance of CRA for patient care, my view of this cohort is that they see CRA as...

- Not at all important (1)
- Slightly important (2)
- Moderately important (3)
- Very important (4)
- Extremely important (5)

Q5 I found teaching CRA to this cohort of students...

- Extremely easy (1)
- Moderately easy (2)
- Slightly easy (3)
- Neither easy nor difficult (4)
- Slightly difficult (5)
- Moderately difficult (6)
- Extremely difficult (7)

Q6 I found supervising this cohort to deliver CRA in clinical practice...

- Extremely easy (1)
- Moderately easy (2)
- Slightly easy (3)
- Neither easy nor difficult (4)
- Slightly difficult (5)
- Moderately difficult (6)
- Extremely difficult (7)

Q7 Please tell us anything else that you feel would be helpful about your experience of working with this cohort of students with regard to CRA

________________________________________________________________

End of Block: Default Question Block
