# Supplementary material for: An evaluation of the use of caries risk/susceptibility assessment in an undergraduate dental curriculum
Source: Front Oral Health. 2024 Jan 29;4:1290713. doi: 10.3389/froh.2023.1290713 (PMC10859480; doi:10.3389/froh.2023.1290713)
Supplement: Supplementary file 3 [file Datasheet3.docx]

Oral Health Risk/Susceptibility Assessment Students' Interview Guide

Start of Block: Default Question Block

Q1 Thinking about your knowledge Oral Health Risk/Susceptibility Assessment in particular Caries Risk/Susceptibility Assessment (CRA/CSA), I think I am……

Q2 Thinking about your competence in carrying out Oral Health Risk/Susceptibility Assessment in particular Caries Risk/Susceptibility Assessment (CRA/CSA), with patients, I think I am …….

Q4 Thinking about confidence in carrying out Oral Health Risk/Susceptibility Assessment in particular Caries Risk/Susceptibility Assessment (CRA/CSA), with patients, I think I am ……

Q3 Thinking about the importance of Oral Health Risk/Susceptibility Assessment in particular Caries Risk/Susceptibility Assessment (CRA/CSA), for patient care, I see Oral Health Risk/Susceptibility Assessment in particular Caries Risk/Susceptibility Assessment (CRA/CSA), as...

Q5 Thinking about how difficult or easy you found learning Oral Health Risk/Susceptibility Assessment in particular Caries Risk/Susceptibility Assessment (CRA/CSA),...

Q6 Thinking about the quality of supervision in delivering Oral Health Risk/Susceptibility Assessment in particular Caries Risk/Susceptibility Assessment (CRA/CSA), in clinical practice...

Q7 Please tell us anything else that you feel would be helpful about your experience with regards to Oral Health Risk/Susceptibility Assessment in particular Caries Risk/Susceptibility Assessment (CRA/CSA),

________________________________________________________________

End of Block: Default Question Block
