# Supplementary material for: An evaluation of the use of caries risk/susceptibility assessment in an undergraduate dental curriculum
Source: Front Oral Health. 2024 Jan 29;4:1290713. doi: 10.3389/froh.2023.1290713 (PMC10859480; doi:10.3389/froh.2023.1290713)
Supplement: Supplementary file 4 [file Datasheet4.docx]

Oral Health Risk/Susceptibility Assessment Teachers' Focus Group Guide

Start of Block: Default Question Block

Q1 Thinking about their knowledge of Oral Health Risk/Susceptibility Assessment in particular Caries Risk/Susceptibility Assessment (CRA/CSA), I think this cohort is...

Q2 Thinking about their carrying out Oral Health Risk/Susceptibility Assessment in particular Caries Risk/Susceptibility Assessment (CRA/CSA), with patients, I think this cohort is...

Q4 Thinking about their carrying out Oral Health Risk/Susceptibility Assessment in particular Caries Risk/Susceptibility Assessment (CRA/CSA), with patients, I think this this cohort is...

Q3 Thinking about the importance of Oral Health Risk/Susceptibility Assessment in particular Caries Risk/Susceptibility Assessment (CRA/CSA), for patient care, my view of this cohort is that they see CRA as...

Q5 Thinking about how difficult or easy, I found teaching Oral Health Risk/Susceptibility Assessment in particular Caries Risk/Susceptibility Assessment (CRA/CSA), to this cohort of students...

Q6 Thinking about how difficult or easy, I found supervising this cohort to deliver Oral Health Risk/Susceptibility Assessment in particular Caries Risk/Susceptibility Assessment (CRA/CSA), in clinical practice...

Q7 Please tell us anything else that you feel would be helpful about your experience of working with this cohort of students with regards to Oral Health Risk/Susceptibility Assessment in particular Caries Risk/Susceptibility Assessment (CRA/CSA),

________________________________________________________________

End of Block: Default Question Block
