# Supplementary material for: An evaluation of the use of caries risk/susceptibility assessment in an undergraduate dental curriculum
Source: Front Oral Health. 2024 Jan 29;4:1290713. doi: 10.3389/froh.2023.1290713 (PMC10859480; doi:10.3389/froh.2023.1290713)
Supplement: Supplementary file 5 [file Datasheet5.docx]

Manuscript order:

| Abbreviation | Full denomination |
| --- | --- |
| MIOC | Minimum Intervention Oral Care |
| CRSA | Caries Risk/Susceptibility Assessment |
| BDS3-20T2 /Group A | Bachelor of Dental Surgery 3 cohort at the end of the academic year 2019-20 |
| BDS4-20T2 /Group B | Bachelor of Dental Surgery 4 cohort at the end of the academic year 2019-20 |
| BDS4-22T1 /Group C | Bachelor of Dental Surgery 4 cohort at the start of the academic year 2021-22, and therefore before the start of the study. |
| BDS4-22T2 /Group D | Bachelor of Dental Surgery 4 cohort at the end of the academic year 2021-22, and therefore after the end of the study. |
| FoDOCS | Faculty of Dental, Oral and Cranio-facial Sciences |
| KCL | King’s College London |
| DEPPA | Denplan PreViser Patient Assessment |
| GSTT) | Guy’s & St Thomas’ Hospital Trust |
| MS | Microsoft |
| MS Teams | Microsoft Teams |
| CPC | Care Planning Clinics |
| GDPR | General Data Protection Regulation |
| IQR | Interquartile Range |
| PRA | Periodontal Risk Assessment |
| CAMBRA | Caries Management by Risk Assessment |
| COVID | Coronovirus |
| NHS | National Health Services |

Alphabetical order:

| Abbreviation | Full denomination |
| --- | --- |
| BDS3-20T2 /Group A | Bachelor of Dental Surgery 3 cohort at the end of the academic year 2019-20 |
| BDS4-20T2 /Group B | Bachelor of Dental Surgery 4 cohort at the end of the academic year 2019-20 |
| BDS4-22T1 /Group C | Bachelor of Dental Surgery 4 cohort at the start of the academic year 2021-22, and therefore before the start of the study. |
| BDS4-22T2 /Group D | Bachelor of Dental Surgery 4 cohort at the end of the academic year 2021-22, and therefore after the end of the study. |
| CAMBRA | Caries Management by Risk Assessment |
| COVID | Coronovirus |
| CPC | Care Planning Clinics |
| CRSA | Caries Risk/Susceptibility Assessment |
| DEPPA | Denplan PreViser Patient Assessment |
| FoDOCS | Faculty of Dental, Oral and Cranio-facial Sciences |
| GDPR | General Data Protection Regulation |
| GSTT | Guy’s & St Thomas’ Hospital Trust |
| IQR | Interquartile Range |
| KCL | King’s College London |
| MIOC | Minimum Intervention Oral Care |
| MS | Microsoft |
| MS Teams | Microsoft Teams |
| NHS | National Health Services |
| PRA | Periodontal Risk Assessment |
